# Associations between therapy experiences and perceived helpfulness of treatment for people with eating disorders

**DOI:** 10.1186/s40337-022-00601-1

**Published:** 2022-06-14

**Authors:** Rahul Mital, Phillipa Hay, Janet E. Conti, Haider Mannan

**Affiliations:** 1 School of Medicine, Western Sydney University, Locked Bag 1797 Penrith South 2715, Sydney, Australia; 2 Translational Health Research Institute, Western Sydney University, Sydney, Australia; 3 South West Sydney Local Health District, Campbelltown, Australia; 4 School of Psychology, Western Sydney University, Sydney, Australia

**Keywords:** Feeding and eating disorders, Therapeutics, Treatment failure, Therapeutic alliance, Patient participation, Multivariate analysis, Regression analysis

## Abstract

**Background:**

Although eating disorders cause significant impairment to an individual’s function, many people disengage from treatment. There is a paucity of literature that focuses on both positive and negative aspects of eating disorder treatment experiences as perceived by the experiencing person. This study aimed to identify the associations between features of therapy with perceived treatment helpfulness across individuals’ most and least helpful treatment experiences.

**Methods:**

An online cross-sectional survey was developed and disseminated, with the data of participants (*n* = 235) being utilised for statistical analyses, including multiple linear regressions.

**Results:**

As predicted, factors in the therapeutic relationship such as the therapist’s ability to instil a sense of hope, provide freedom of choice, understand the person, and address participant concerns had significant explanatory value in perceived treatment helpfulness. Contrary to our hypothesis, change being retrospectively identified as important or possible by the participant did not have a high degree of relation. These outcomes highlighted the significance of the therapeutic relationship in governing positive treatment experiences and responses. The results also suggested motivation to change when commencing treatment may not be strongly related to perceived treatment helpfulness and support further exploration.

**Supplementary Information:**

The online version contains supplementary material available at 10.1186/s40337-022-00601-1.

## Plain English Summary

Eating disorders can cause a large impact to the lives of those living with them and their loved ones. There is a need for research from the perspective of the individual with lived eating disorder experience to shape treatment for these conditions. This study aimed to address this need by investigating features of therapy and how they impacted the overall perception of treatment helpfulness. It was found that therapist related factors, such as providing freedom of choice around change and instilling hope, accounted for a large contribution to helpfulness. It was also found that motivation may not necessarily have to be high when commencing treatment to result in better ratings of treatment helpfulness.

**Supplementary Information:**

The online version contains supplementary material available at 10.1186/s40337-022-00601-1.

## Background

Despite the increased prevalence [1] and disease burden [2] of eating disorders (EDs), coupled with mortality rates almost twice as high on average, and 5.68 times higher in anorexia nervosa (AN) [3], many people do not seek treatment [4] and for those who do, attrition rates are high [5]. Thus, there is ongoing need for research into understanding treatment from the perspective of those with a lived ED experience, particularly the discernment of what works, and for whom, in ED psychotherapy treatments. This research has scope to provide direction into enhancing current treatment approaches and therapeutic engagement to improve treatment outcomes and reduce the sequelae of EDs.

An important factor that contributes to treatment engagement and enhances outcomes and experience, is a collaborative therapeutic approach where therapists take into account the preferences of the experiencing person [6]. It enables the conceptualisation of a shared treatment framework that facilitates a sense of personal agency within the individual. A cross-national investigation for ED services treatment demonstrated that major barriers to treatment involved the fear of not being able to voice concerns, and not having treatment choices and autonomy [7]. This concern was echoed in a study where individuals with a former AN diagnosis identified the importance of collaboration, including in the formulation of shared goals and in the definition of recovery [8].

There is a paucity of meta-analyses focusing specifically on client preferences and treatment outcomes in eating disorders, however, there have been several meta-analyses that have explored more broadly the influence of accommodation of client preferences on psychotherapy outcomes [9]. For example, a meta-analysis of pooled data from 35 studies on psychotherapy outcomes found that when patient preferences were accommodated that the odds ratio was 0.59 for treatment attrition and the Cohen’s d was 0.31 for associated treatment outcomes [10]. This result was consistent across client characteristics and treatment duration [11]. A further meta-analysis that utilised the data of 186 comparative trials found that both treatment refusal and premature treatment termination were linked with a non-preferred treatment modality [12]. This finding is consistent with Lindhiem, Bennett [13]’s meta-analysis, which found that clients who were involved in the decision-making process of their treatment had significantly higher rates of treatment completion. In light of this research, Swift et al. [14] has postulated that accommodating for preference may enhance motivation and thus be the explanation for better treatment outcomes. To increase understanding of the role of collaborative care in the treatment of eating disorders, further research is indicated.

Therapeutic alliance embodies the relational bond and the “goodness of fit” between the individual and therapist that is established through collaborative therapeutic work, mutual trust, empathy and hope [15]. A strong therapeutic alliance has been shown to be a positive predictive factor in improvement for ED symptomology [16–22]. Less understood is the relationship between the quality of the therapeutic alliance and perceived treatment helpfulness and its contribution to a recovery-oriented, person-centred approach as recommended in guidelines [23]. In broader psychotherapy contexts, the therapeutic relationship has been found to account for at least as much of client improvement as treatment method selection [24]. In a qualitative metasynthesis, individuals with a lived experience of AN preferred treatments in which the therapist treated them holistically and believed in their ability to re-establish sustainable identities beyond their diagnosis [25]. This represents a core component of the therapeutic relationship and is an under-researched area in the field: the examination of the treating therapist’s ability to instil a sense of hope within the individual.

Motivation to change implies the intention or belief to change one's behaviour, and is another aspect that impacts treatment engagement. Keski-Rahkonen and Tozzi [26] postulated that the value of treatment is conditional on the person’s individual desire to change. Furthermore, systematic reviews [5, 27] found that lower levels of motivation are associated with higher rates of premature treatment discontinuation and chronicity of disease. When an individual is not ready to engage in treatment due to low motivation, adverse treatment outcomes may ensue. Although numerous studies [28–31] highlight the importance of motivation in determining treatment outcomes, there is a lack of empirical research on the importance of the individual’s intrinsic motivation at the time of commencing treatment with later perceived treatment helpfulness.

Dawson, Rhodes [32] suggests that intervention research has generally adopted the medical model, focussed on symptom amelioration. Newer evidence has suggested that there should be a focus on the process of treatment [25] from the perspective of the treatment recipient and overall perceived helpfulness as the outcome of interest [16]. A randomised controlled trial (RCT) that explored the subjective evaluation of outpatient treatment for AN, highlighted the importance for future research to explore predictors including therapeutic alliance, therapists’ characteristics, expectations of therapy and self-evaluated therapy success [33]. The same study concluded that objective treatment outcome in adolescent outpatients did not accurately predict treatment satisfaction, further emphasising the need for the present study. Most understanding of ED treatment efficacy is based on RCTs in specialist treatment environments, while little is known about how treatment is experienced more broadly in real world settings. There is limited literature regarding an integrated understanding of factors related to perceptions of therapy experiences, and putative treatment engagement. In sum, to further the development of evidence-based practice for eating disorders, it is relevant to explore therapy-related determinants of perceived treatment helpfulness.

Therefore, the present study was designed to explore the relative strength of associations between determinants of participants’ eating disorder treatment experiences with overall perceived helpfulness of treatment through describing features associated with their most and least helpful treatment experiences. We hypothesised that the following would be associated with positively, and if absent, negatively perceived treatment helpfulness: (1) a collaborative treatment approach that provided freedom of choices around change, (2) a therapist who was perceived to understand the participant and address their concerns, (3) a therapist who was perceived to instil hope, and (4) if change was retrospectively identified as important or possible to the individual.

## Methods

### Design

The present study is a cross-sectional quantitative analysis of self-reported eating disorder treatment experiences. An online questionnaire, *Eating Disorders Treatment Experiences Survey* was disseminated through advertising on social media via eating disorder Facebook sites in Australia, New Zealand, USA, and UK. Due to snowball sampling, participants from other countries also participated and were included in the study. The online survey was hosted by Qualtrics.com and completed anonymously. After being presented with information regarding the survey, all participants endorsed consent.

### Participants

Participants were included if they reported that they were “currently receiving treatment” or had “received treatment but not currently” for an ED. Participants were excluded for incomplete responses in the “most positive treatment experience” or “least positive treatment experience” items. Using a G*Power analysis [34], the sample (n = 235) was considered to have ample statistical power for hypothesis testing at the 0.40 and 0.20 level.

### Outcome measures

#### Demographic features

Participants’ age, weight, and height (to calculate body mass index, BMI, kg/m^2^), country of residence, marital status, gender, and diagnosis type were self-reported.

#### Eating disorder examination questionnaire short (EDE-QS)

The EDE-QS [35] is a 12-item questionnaire in which increasing scores correspond with worsening severity of ED symptomology. The EDE-QS has demonstrated good discriminatory power compared to other instruments for ED screening when participants score 15 or higher (range 0–36) [36]. Gideon, Hawkes [35] found the EDE-QS to demonstrate a high internal consistency with Cronbach’s α = 0.913. The present study demonstrated a high internal consistency, Cronbach’s α = 0.904, N = 12.

#### Hospital anxiety and depression scale (HADS)

The HADS [37] is a brief questionnaire where 7 items rate anxiety and depression scores respectively. Scores range from 0 to 21 for each subset with increasing scores reflecting increased severity of disease. A 28-study systematic review and meta-analysis [38] found that scores of 10 or 11 represented a sensitivity of 0.80 for mental health disorders. In the present sample, for the anxiety subset Cronbach’s α = 0.857 and for the depression subset α = 0.845.

#### Least helpful/most helpful therapy items

Participants completed a series of items scored on Likert scales (− 50 to 50, range = 100). These were adapted from *Session Rating Scal*e [39] regarding individual and therapy related factors in participants’ most helpful and least helpful treatment experiences (see Additional file 1: Appendix). For the present study, the two items related to treatment approach, that is, “took into consideration my treatment preference” and “gave me freedom to make my own choices around change” were averaged to form the first explanatory variable [1]. Similarly, “I felt understood by the therapist” and “the therapist addressed my concerns” were averaged to produce the second explanatory variable [2]. “The therapist did instil hope for recovery” was used as the third explanatory variable [3]. “When I sought this treatment, I thought change was important or possible” was used as the fourth explanatory variable [4]. The criterion (dependent) variable of interest was the item “Overall, how helpful do you think your eating disorder treatment was?”. Internal consistency was high for most helpful and least helpful therapy items, α = 0.926 and α = 0.868 respectively. Finally, participants’ mood at the time of each treatment experience was recorded on a 4-point scale, in which a score of 1 was extremely negative and 4 was positive.

### Statistical analyses

#### Demographic and clinical features

Descriptive analyses for demographic and clinical features of the participants were performed using SPSS Version 27.0 [40].

#### Correlations

Due to violations of normality, Spearman’s non-parametric bivariate correlation analyses were conducted to investigate the relationships between the hypothesized variables and the perceived ratings of treatment helpfulness. SPSS Version 27.0 was used [40].

#### Multiple linear regressions

Once all assumptions had been met, multiple linear regression models were fitted for dependent variables: *MH Helpfulness* (overall perceived treatment helpfulness for most helpful treatment) and *LH Helpfulness* (overall perceived helpfulness for least helpful treatment). For both most helpful treatment (n = 229) and least helpful treatment models (n = 225), the determinants were: (1) treatment considered the participant’s preferences and provided freedom of choices around change, (2) therapist was perceived to have understood the participant and addressed their concerns, (3) therapist rating to have instilled a sense hope and (4) whether change was retrospectively identified as important or possible. All statistical tests for the independent variables were performed at the level of 5% significance (alpha = 5%). We checked multi-collinearity among the four explanatory variables using the variance inflation factor (VIF) and found that none were greater than 10. Thus, indicating lack of multicollinearity.

#### Exploratory analysis

The dependent variable, *MH Helpfulness,* was negatively skewed, therefore a cubic transformation was required to have the residuals follow a normal distribution [41]. The coefficient of skewness in the untransformed model was -1.156 and was reduced to − 0.369 following cubic transformation. The dependent variable, *LH Helpfulness,* was positively skewed and consequently, a squared root transformation was required [41]. The coefficient of skewness in the untransformed model was 1.108 and following the squared root transformation was reduced to 0.377. We also attempted log and reciprocal transformations due to their relative ease of interpretation. Using these transformations however, the residuals of *LH Helpfulness* did not follow normal distribution and thus failed to achieve our objective. The residuals of both transformed models were found to be normally distributed using a Shapiro–Wilk test [42]. To check for homoscedasticity of variance of these models, we performed the Breusch-Pagan test which supported the homoscedasticity assumption [43]. The median scores for overall perceived treatment helpfulness, a collaborative treatment approach that provided freedom of choices around change, a therapist who was perceived to understand the participant and address their concerns, a therapist who was perceived to instil hope, and if change was retrospectively identified as important or possible to the individual, were compared between most helpful and least helpful treatment experiences using the Wilcoxon Signed Rank Test.

Following the transformations of the dependent variables, SAS version 9.4 was used to perform multiple linear regressions on each of the models [44]. To find the regression coefficients and their 95% confidence intervals for *MH Helpfulness* on the original scale, we back-transformed the results of the multiple linear regressions. The estimated standard errors were back-transformed using the Delta method [45], for which R 3.6.6, package ‘msm’ [46] was used. For *LH Helpfulness*, it is not recommended to back-transform to the original scale due to the nature of the squared root transformation. Negative predictions are squared and become positive values, thus introducing non-monotonicity in the model. Therefore, the transformed model was used to evaluate significant predictors, rather than a back-transformed model. For a back-transformed model in the case of *MH Helpfulness*, p-values cannot be calculated on the original scale. Thus, for uniformity, statistical significance for all determinants in both models was determined using the 95% confidence interval of the partial regression coefficient.

## Results

### Demographic and clinical features of participants

A total of 235 participants were included in the present study; 218 female, 2 male, 15 non-binary, median age 22 (IQR 19–28), BMI: median 21.89 (IQR: 19.57–28.73). 68.3% of participants reported a diagnosis of an eating disorder had been made within the preceding seven years. The countries of residence were predominantly developed and of English-speaking background: 59 Australia or New Zealand, 47 UK, 112 USA or Canada, 5 other, 12 missing. Participants had high levels of anxiety (median 13, IQR 10–15) however lower levels of depression (median 7, IQR 4–11). The median EDE-QS score was 21 (IQR 22–36), which is at a likely clinical level of severity in ED symptomology. All other descriptive statistics for demographic and clinical features of participants are contained within Table 1.

**Table 1:** Demographic and clinical features of the participants

Feature	Statistic	
	N	%
*Marital status*		
Single	135	57.4
In a relationship (not De-Facto/Married)	50	21.3
De-Facto/Married	29	12.3
Separated/Divorced/Widowed	9	3.8
Missing	12	5.1
*Total*	235	
*Self-reported eating disorder diagnosis*		
Anorexia Nervosa	157	66.8
Bulimia Nervosa	18	7.7
Binge Eating Disorder	9	3.8
Orthorexia Nervosa	1	0.4
Multiple Eating Disorders	17	7.2
Eating Disorder not otherwise specified (EDNOS)	29	12.3
Missing	4	1.7
*Total*	235	
*Treatment history*		
Currently receiving treatment for an eating disorder	90	38.3
Received treatment in the past but not currently	145	61.7
*Total*	235	
*Mood recollection at time of least helpful treatment*		
Extremely negative	96	40.9
Negative	77	32.8
Neither positive nor negative	47	20
Positive	14	6
Missing	1	0.04
*Total*	235	
*Mood recollection at time of most helpful treatment*		
Extremely negative	52	22.1
Negative	63	26.8
Neither positive nor negative	77	32.8
Positive	43	18.3
Missing	0	0
*Total*	235	
*Treatment modality for least helpful treatment*		
Inpatient	60	25.5
Group therapy	18	7.7
CBT	21	8.9
Family based therapy	18	7.7
Other therapy	38	16.2
Dietitian	15	6.4
Pharmacotherapy	8	3.4
Other	25	10.6
Partial hospitalisation/day program	22	9.4
Unknown	10	4.3
*Total*	235	
*Treatment modality for most helpful treatment*		
Inpatient	25	10.6
Group therapy	4	1.7
CBT	20	8.5
Family based therapy	4	1.7
Other therapy	70	29.8
Dietitian	17	7.2
Pharmacotherapy	4	1.7
Other	48	20.4
Partial hospitalisation/day program	31	13.2
Unknown	12	5.1
*Total*	235	

The median scores for all variables of interested were compared between most helpful and least helpful treatment experiences using the Wilcoxon Signed Rank Test. All were significantly different favouring the most helpful treatment experience (standardised test statistic, P < 0.001 for all). Overall perceived treatment helpfulness (Standardised test statistic Z = 12.779), a collaborative treatment approach that provided freedom of choices around change (Z = 12.168), a therapist who was perceived to understand the participant and address their concerns (Z = 12.470), a therapist who was perceived to instil hope (Z = 10.789), and if change was retrospectively identified as important or possible to the individual (Z = 4.287).

### Correlations between four explanatory variables

Results from the Spearman’s non-parametric bivariate correlation analysis between the four explanatory variables and perceived treatment helpfulness are presented in Table 2. All variables were found to have statistically significant positive correlations at the 0.05 level (2-tailed) between determinants and perceived helpfulness except for the fourth explanatory variable, that is, change retrospectively identified as important or possible in the least helpful treatment experience.

**Table 2:** Associations between overall most and least helpful therapy experiences and therapist/treatment qualities

Therapist/Treatment qualities	MH Overall helpfulness	LH overall helpfulness
*Spearman’s rho (p)*		
Treatment approaches that took into account participant treatment preferences and provided freedom of choice around change	0.543* (< 0.005)	0.537* (< 0.005)
Therapist perceived to have understood them and addressed their concerns	0.641* (< 0.005)	0.654* (< 0.005)
Therapist perceived to have instilled hope	0.718* (< 0.005)	0.606* (< 0.005)
Change retrospectively identified as important or possible	0.179* (0.006)	0.128 (0.052)

^*^Correlation significant at the 0.05 level (2-tailed)

### Multiple linear regressions

The predictor variables were ranked in order of contribution (R^2^) to perceived treatment helpfulness. That is, R^2^ represents the proportion of variance for overall perceived treatment helpfulness as explained by each predictor variable. Partial regression coefficient (B), standard error (se) and the 95% confidence interval (CI) in Table 3 and 4 for *MH Helpfulness* and *LH Helpfulness* respectively.

**Table 3:** Multiple linear regression of the back-transformed Most Helpful treatment model

Independent Variable	B (se)	95% CI	R^2^
Treatment approaches that took into account participant treatment preferences and provided freedom of choice around change (1)	12.468* (1.702)	7.205 to 15.152	**0.304**
Therapist perceived to have understood them and addressed their concerns (2)	10.479 (3.565)	− 10.517 to 15.132	**0.171**
Therapist perceived to have instilled hope (3)	19.621* (0.936)	17.570 to 21.315	**0.130**
Change retrospectively identified as important or possible (4)	7.257 (3.234)	− 8.549 to 11.158	**0.001**

^*^Statistically significant result

**Table 4:** Multiple linear regression of the squared root transformed Least Helpful treatment model

Independent Variable	B (se)	95% CI	R^2^
Therapist perceived to have understood them and addressed their concerns (2)	0.318* (0.070)	0.180 to 0.456	**0.195**
Treatment approaches that took into account participant treatment preferences and provided freedom of choice around change (1)	0.084 (0.073)	− 0.060 to 0.229	**0.132**
Therapist perceived to have instilled hope (3)	0.217* (0.057)	0.106 to 0.329	**0.042**
Change retrospectively identified as important or possible (4)	0.025 (0.043)	− 0.060 to 0.110	**0.001**

^*^Statistically significant result

The adjusted R^2^ of the back-transformed, most helpful model was 0.5995; that is, 59.95% of the total variance in *MH Helpfulness* was explained by the four explanatory variables after adjusting for their number. The explanatory variable, a therapist who was perceived to understand the participant and address their concerns and the explanatory variable, change being retrospectively identified as important or possible, were not found to be statistically significant albeit positively correlated with the criterion variable. The remaining explanatory variables were statistically significant and positively correlated with the criterion variable. The partial regression coefficient is interpreted as in the following example: for a therapist who was perceived to have instilled hope [3] B was 19.621, that is, for a one unit increase in this variable, *MH Helpfulness* increased by 19.621 units.

The adjusted R^2^ of the transformed, least helpful model was 0.358, that is, 35.8% of the variance in squared root of *LH Helpfulness* was accounted for by the four explanatory variables after adjusting for their number. The explanatory variable, a collaborative treatment approach that provided freedom of choices around change and the explanatory variable, change being retrospectively identified as important or possible, were not found to be statistically significant albeit positively correlated with the criterion variable. The remaining explanatory variables were statistically significant and positively correlated with the criterion variable. B in this model should be interpreted with caution. For a treatment that considered treatment preferences and provided freedom of choice around change [1], B was estimated to be 0.084, which means that for a one-unit increase in this variable, the squared root of *LH Helpfulness* increased by 0.084 units.

## Discussion

The primary objective of this study was to assess participants’ most helpful and least helpful treatment experiences through analyses of therapy related factors rated against perceived treatment helpfulness. Results from the Spearman’s correlation analysis and subsequent multiple linear regression models, confirmed the posited relationship between therapist related factors and positive perceptions of treatment.

A higher proportion of participants’ treatment modality was inpatient treatment in the least helpful treatment model. This may suggest that when autonomy is removed and individual preferences are less accommodated in a more restrictive treatment setting with participants with more severe ED symptoms, they are less likely to perceive treatment as helpful. In the most helpful treatment model outpatient therapy in many forms dominated treatment modality. When recalling their most helpful treatment, responses demonstrated that a treatment approaches that took into account participants preferences and facilitated room for change, thus providing a greater degree of autonomy, made the most significant contribution to overall perceived treatment helpfulness. In the least helpful treatment model, the strongest analysed moderator was a therapist who was perceived to understand the participant and address their concerns. This highlights the impact that bespoke, individualised treatment, or lack thereof, has in participants’ least helpful treatment experiences. In both models, the impact of the therapist’s ability to instil a sense of hope in overall perceived treatment helpfulness was affirmed. Contrary to our expectation, change being retrospectively identified as important or possible was not strongly associated with perceived treatment helpfulness.

Results from this study align with those of previous literature and support the importance of therapeutic alliance in predicting a positive treatment response [16–19]. This study differs however, in that the criterion variable was perceived treatment helpfulness in contrast to objective ED symptom cure. It was expected that participants who perceived their therapist as understanding and addressed their concerns were more likely to view their treatment as positive, forming an integral part of the therapeutic alliance [22]. Similarly, as hypothesised, a collaborative approach to treatment with a high degree of importance given to individual preferences and freedom was found to demonstrate positive effects on perceived helpfulness, also consistent with existing literature [7, 8].

A major discordant finding in our study was a lack of strong association between value or belief in change when initiating treatment and positive or negative perceptions of treatment helpfulness. Furthermore, in the most helpful treatment model, this explanatory variable represented the lowest contribution to perceived helpfulness. This contradicts prior studies that postulated an individual’s intrinsic value for change was fundamental to aiding recovery [26, 47–49]. This may be explained by motivation being continuously engendered throughout treatment, through means such as motivational interviewing [50] rather than being a prerequisite for treatment. Dare, Eisler [21] suggested that the therapist should synchronise their personal experience with the participant to promote recovery. This is supported in our findings by the comparatively high contribution to perceived helpfulness attributed to therapeutic alliance, particularly the therapist’s ability to instil a sense of hope. It is also important to note that people with AN, who make up the majority of the present participants (66.8%), tend to have an egosyntonic view of their illness [31]. Thus, importance or belief in change may have been less valued by these participants than people with other mental health conditions.

There were several limitations identified in the present study. In the present sample, participants were inclined to be younger, female, from a higher-income and predominantly English-speaking country, and have a primary diagnosis of AN. This selection bias is likely a result of the online nature of advertising on social media used for recruitment and limits the generalisability of our findings to broader ED populations. Further, because of the international recruitment of the survey and to reduce participant assessment burden, ethnicity or race were not asked until the end of the questionnaire and therefore under-reported with few participants completing this final demographic section of the survey. Moreover, all items used for the statistical correlation and regression analyses were self-reported and retrospective, rendering them susceptible to recall bias. This may have had several impacts, such as the severity of the participant’s negative mood at the time of most helpful treatment being diminished and vice versa. It is also difficult to make assertions regarding efficacy of different treatment modalities in the models as these too were self-reported. Further, measurement of perceived instilled hope, motivation and possibility for change, and perceived treatment helpfulness were single-item scales, diminishing our ability to wholly capture these constructs. Additionally, due to the statistical complications encountered with transformations, the partial regression coefficients from the multiple linear regression analyses could not be compared across the two models. Therapy features of interest have been applied across all treatments, which may confound results in certain scenarios. For example, in inpatient treatment, autonomy may be reduced when compared to an outpatient setting, therefore freedom and preferences around change would be skewed towards lower ratings. The study is also cross-sectional and thus findings are associative, and causality cannot be inferred. Finally, it is important to acknowledge the complexities of experiences of therapy and nature of the therapeutic relationship across the spectrum of mental health disorders and different settings which can give rise to different findings regarding the impact of the therapeutic alliance.

To the authors’ knowledge, this study has been the first of its kind to perform a quantitative analysis on both positive and negative treatment experiences for individuals with lived ED experience. As such, multiple linear regression analyses were able to rank variables and compare their relative importance in helpful and unhelpful experiences of therapy. Furthermore, this study specifically explored predictors recommended by Jaite, Pfeiffer [33], including therapeutic alliance, therapists’ characteristics, and self-evaluated therapy success. This study also addresses the paucity of research in therapeutic success measured by participant experience rather than therapist-identified goals [16]. Moreover, there was a large sample size included with ample statistical power, largely due to the international recruitment and community sampling that was achieved in this study. This paper also provides insight into the relative importance of motivation when commencing treatment, a consideration incompletely explained by previous literature [28–31].

## Conclusions

It is anticipated that this study will provide directions for future research and inform a clinically appropriate approach to treatment to improve the individual’s satisfaction, treatment completion and clinical outcomes. This paper demonstrates the value of enabling treatment preferences and freedom of choice around change in enhancing perceived treatment helpfulness. Further, the role of the therapeutic alliance through understanding and addressing concerns and instilling hope was clarified to have significant associations with heightened perceptions of treatment. The assumption that motivation needs to be high for perceived change to occur was questioned as it was found to be relatively less strongly associated with perceived treatment helpfulness when compared to other therapy related factors. The findings also suggest that irrespective of the motivation level at the start of therapy, it is appropriate to work with the person to enhance motivation throughout the course of treatment. It may be also appropriate to modify the pace of treatment if the level of motivation is low.

It is recommended that research in this field is continued, particularly for prospective longitudinal studies to establish causality of determinants for treatment helpfulness. There should be a focus on sampling to include non-AN and male participants to assess the fidelity of our findings against broader ED populations. Finally, there should be a focus on analysis of objective ED symptomology resolution alongside perceived treatment helpfulness. There are also a number of moderating factors outside of therapy related factors that could be explored in future publications.

## Supplementary Information

**Additional file 1: Appendix.** Survey measures.

## Data Availability

Data are available from the authors upon request.

## Abbreviations

ED: Eating disorder
DSM-5: Diagnostic and statistical manual of mental disorders
AN: Anorexia nervosa
BN: Bulimia nervosa
BED: Binge eating disorder
ARFID: Avoidant/restrictive food intake disorder
OSFED: Other specified feeding or eating disorder
UFED: Unspecified feeding or eating disorder
DALY: Disability adjusted life years
OR: Odds ratio
CI: Confidence interval
RCT: Randomized control trial
EDE-QS: Eating disorder examination–questionnaire short
MH Helpfulness: Dependent variable: perceived treatment helpfulness for most helpful treatment
LH Helpfulness: Dependent variable: perceived treatment helpfulness for least helpful treatment
IQR: Interquartile range
B: Partial regression coefficient
se: Standard error
R^2^: Coefficient of determination

## References

[1] Hay PJ, Mond J, Buttner P, Darby A (2008). Eating disorder behaviors are increasing: findings from two sequential community surveys in South Australia. PLoS ONE.

[2] Galmiche M, Déchelotte P, Lambert G, Tavolacci MP (2019). Prevalence of eating disorders over the 2000–2018 period: a systematic literature review. Am J Clin Nutr.

[3] Arcelus J, Mitchell AJ, Wales J, Nielsen S (2011). Mortality rates in patients with anorexia nervosa and other eating disorders. A meta-analysis of 36 studies. Arch Gen Psychiatry.

[4] Hart LM, Granillo MT, Jorm AF, Paxton SJ (2011). Unmet need for treatment in the eating disorders: a systematic review of eating disorder specific treatment seeking among community cases. Clin Psychol Rev.

[5] Fassino S, Pierò A, Tomba E, Abbate-Daga G (2009). Factors associated with dropout from treatment for eating disorders: a comprehensive literature review. BMC Psychiatry.

[6] Norcross JC (2011). Psychotherapy relationships that work evidence-based responsiveness.

[7] Escobar-Koch T, Banker JD, Crow S, Cullis J, Ringwood S, Smith G (2010). Service users' views of eating disorder services: an international comparison. Int J Eat Disord.

[8] Darcy AM, Katz S, Fitzpatrick KK, Forsberg S, Utzinger L, Lock J (2010). All better? How former anorexia nervosa patients define recovery and engaged in treatment. Eur Eat Disord Rev.

[9] Swift JK, Callahan JL, Cooper M, Parkin SR, Swift JK, Callahan JL, Cooper M, Parkin SR (2019). Preferences. Psychotherapy relationships that work: evidence-based therapist responsiveness.

[10] Swift JK, Callahan JL, Vollmer BM, Norcross JC (2011). Preferences. Psychotherapy relationships that work: evidence-bases responsiveness.

[11] Swift JK, Callahan JL, Ivanovic M, Kominiak N (2013). Further examination of the psychotherapy preference effect: a meta-regression analysis. J Psychother Integr.

[12] Swift JK, Greenberg RP, Tompkins KA, Parkin SR (2017). Treatment refusal and premature termination in psychotherapy, pharmacotherapy, and their combination: a meta-analysis of head-to-head comparisons. Psychother Theory Res Pract Train.

[13] Lindhiem O, Bennett CB, Trentacosta CJ, McLear C (2014). Client preferences affect treatment satisfaction, completion, and clinical outcome: a meta-analysis. Clin Psychol Rev.

[14] Swift JK, Mullins RH, Penix EA, Roth KL, Trusty WT (2021). The importance of listening to patient preferences when making mental health care decisions. World Psychiatry.

[15] Bordin ES (1979). The generalizability of the psychoanalytic concept of the working alliance. Psychother Theor Res Pract.

[16] de la Rie S, Noordenbos G, Donker M, van Furth E (2008). The quality of treatment of eating disorders: a comparison of the therapists' and the patients' perspective. Int J Eat Disord.

[17] Pannekoek L, Byrne S, Fursland A (2015). Finding modifiable predictors of treatment dropout: the role of the therapeutic alliance, readiness to change and acceptance of treatment approach. J Eat Disord.

[18] Stiles-Shields C, Touyz S, Hay P, Lacey H, Crosby RD, Rieger E (2013). Therapeutic alliance in two treatments for adults with severe and enduring anorexia nervosa. Int J Eat Disord.

[19] Tozzi F, Sullivan PF, Fear JL, McKenzie J, Bulik CM (2003). Causes and recovery in anorexia nervosa: the patient's perspective. Int J Eat Disord.

[20] Fogarty S, Ramjan LM (2016). Factors impacting treatment and recovery in Anorexia Nervosa: qualitative findings from an online questionnaire. J Eat Disord.

[21] Dare C, Eisler I, Russell G, Treasure J, Dodge L (2001). Psychological therapies for adults with anorexia nervosa: randomised controlled trial of out-patient treatments. Br J Psychiatry.

[22] Ackerman SJ, Hilsenroth MJ (2003). A review of therapist charcteristics and techniques positively impacting the therapeutic alliance. Clin Psychol Rev.

[23] Hay P, Chinn D, Forbes D, Madden S, Newton R, Sugenor L (2014). Royal Australian and New Zealand College of psychiatrists clinical practice guidelines for the treatment of eating disorders. Aust N Z J Psychiatry.

[24] Norcross JC, Wampold BE (2018). A new therapy for each patient: Evidence-based relationships and responsiveness. J Clin Psychol.

[25] Conti JE, Joyce C, Hay P, Meade T (2020). Finding my own identity: a qualitative metasynthesis of adult anorexia nervosa treatment experiences. BMC Psychol.

[26] Keski-Rahkonen A, Tozzi F (2005). The process of recovery in eating disorder sufferers' own words: an Internet-based study. Int J Eat Disord.

[27] Abdelbaky G, Hay P, Touyz S (2013). A systematic review of treatment attrition in anorexia nervosa. J Eat Disord.

[28] Carter O, Byrne S, Allen K, Fursland A (2014). Readiness and motivation to change in the treatment of adults with anorexia nervosa: a case series. J Eat Disord.

[29] Fitzpatrick ME, Weltzin T (2014). Motivation for change as a predictor of eating disorder treatment outcomes using a brief self-report YBC-EDS in a residential eating disorder population. Eat Behav.

[30] Sansfaçon J, Gauvin L, Fletcher É, Cottier D, Rossi E, Kahan E (2018). Prognostic value of autonomous and controlled motivation in outpatient eating-disorder treatment. Int J Eat Disord.

[31] Nordbø RH, Gulliksen KS, Espeset EM, Skårderud F, Geller J, Holte A (2008). Expanding the concept of motivation to change: the content of patients' wish to recover from anorexia nervosa. Int J Eat Disord.

[32] Dawson L, Rhodes P, Touyz S (2014). Doing the impossible: the process of recovery from chronic anorexia nervosa. Qual Health Res.

[33] Jaite C, Pfeiffer A, Pfeiffer E, Thurn C, Bierbaum T, Winter SM (2020). Subjective evaluation of outpatient treatment for adolescent patients with anorexia nervosa. Eat Weight Disord.

[34] Faul F, Erdfelder E, Lang AG, Buchner A (2007). G*Power 3: a flexible statistical power analysis program for the social, behavioral, and biomedical sciences. Behav Res Methods.

[35] Gideon N, Hawkes N, Mond J, Saunders R, Tchanturia K, Serpell L (2016). Development and psychometric validation of the EDE-QS, a 12 item short form of the eating disorder examination questionnaire (EDE-Q). PLoS ONE.

[36] Prnjak K, Mitchison D, Griffiths S, Mond J, Gideon N, Serpell L (2020). Further development of the 12-item EDE-QS: identifying a cut-off for screening purposes. BMC Psychiatry.

[37] Zigmond AS, Snaith RP (1983). The hospital anxiety and depression scale. Acta Psychiatr Scand.

[38] Vodermaier A, Millman RD (2011). Accuracy of the hospital anxiety and depression scale as a screening tool in cancer patients: a systematic review and meta-analysis. Support Care Cancer.

[39] Duncan B, Miller S, Sparks J, Claud DA, Reynolds L, Brown J (2003). The session rating scale: preliminary psychometric properties of a "working" alliance measure. J Brief Ther.

[40] IBM Corp. IBM SPSS Statistics for Macintosh, Version 27.0. Armonk, NY2020.

[41] Box GEP, Cox DR (1964). An analysis of transformations. J Roy Stat Soc Ser B (Methodol).

[42] Shapiro SS, Wilk MB (1965). An analysis of variance test for normality (complete samples)†. Biometrika.

[43] Breusch TS, Pagan AR (1979). A Simple test for heteroscedasticity and random coefficient variation. Econometrica.

[44] SAS Institute Inc. SAD 9.4. Cary, NC 2013.

[45] Kelley TL (1928). Crossroads in the mind of man; a study of differentiable mental abilities.

[46] Jackson C (2001). Multi-state models for panel data: the msm package for R. J Stat Softw.

[47] Gonidakis F, Karapavlou D-A, Jauregui-Lobera I (2017). The patient’s perspective: exploring factors that contribute to recovery from eating disorders. Eating disorders-a paradigm of the biopsychosocial model of illness.

[48] Hay PJ, Cho K (2013). A qualitative exploration of influences on the process of recovery from personal written accounts of people with anorexia nervosa. Women Health.

[49] Smith KE, Mason TB, Leonard RC, Wetterneck CT, Smith BER, Farrell NR (2018). Affective predictors of the severity and change in eating psychopathology in residential eating disorder treatment: the role of social anxiety. Eat Disord.

[50] Miller WR, Rollnick S (2013). Motivational interviewing: helping people change.

