# Supplementary figures and images for: Associations between therapy experiences and perceived helpfulness of treatment for people with eating disorders
Source: J Eat Disord. 2022 Jun 14;10:80. doi: 10.1186/s40337-022-00601-1 (PMC9199215; doi:10.1186/s40337-022-00601-1)

## ***Appendix***

Appendix : survey measures


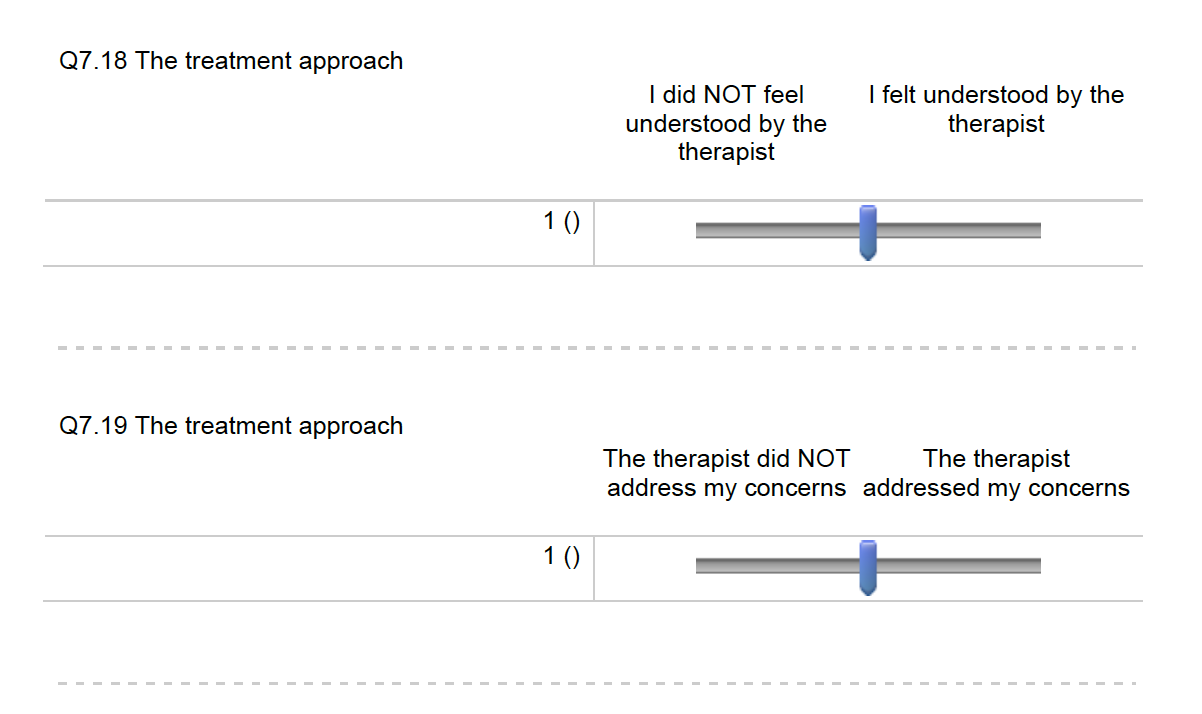

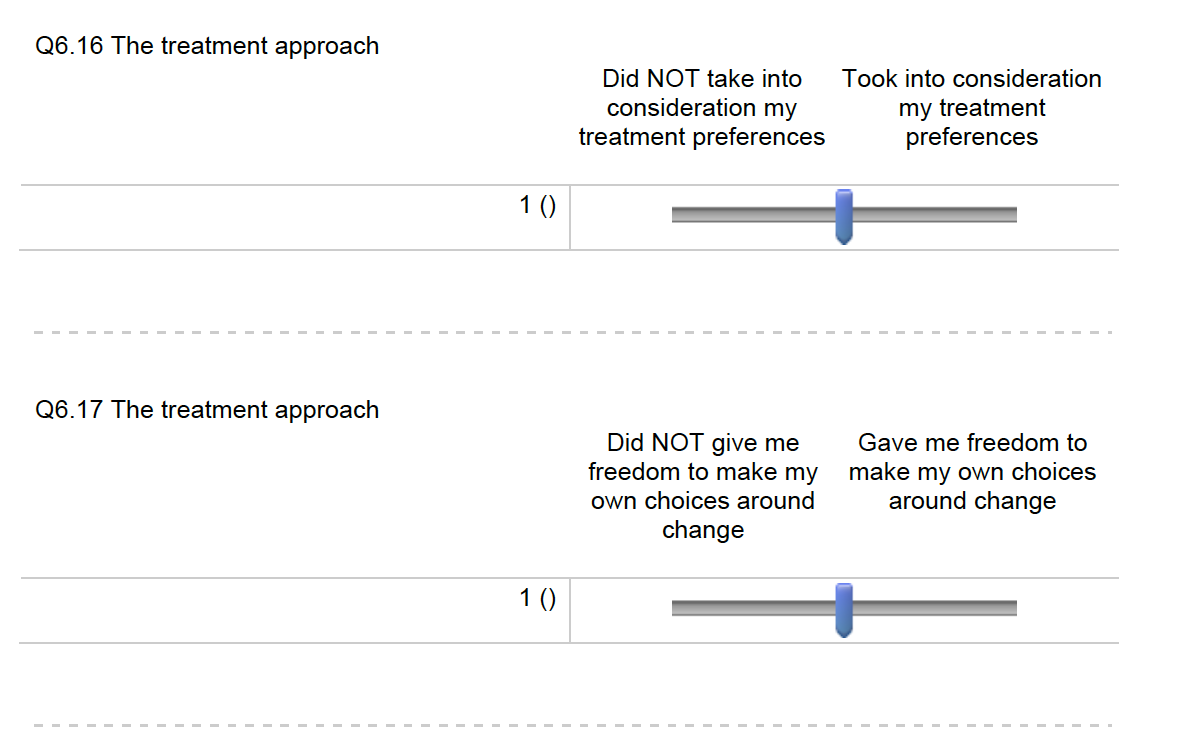


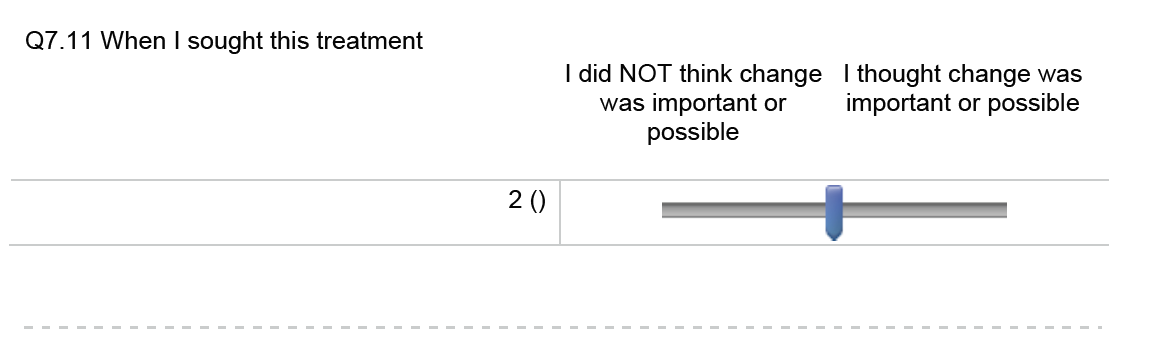

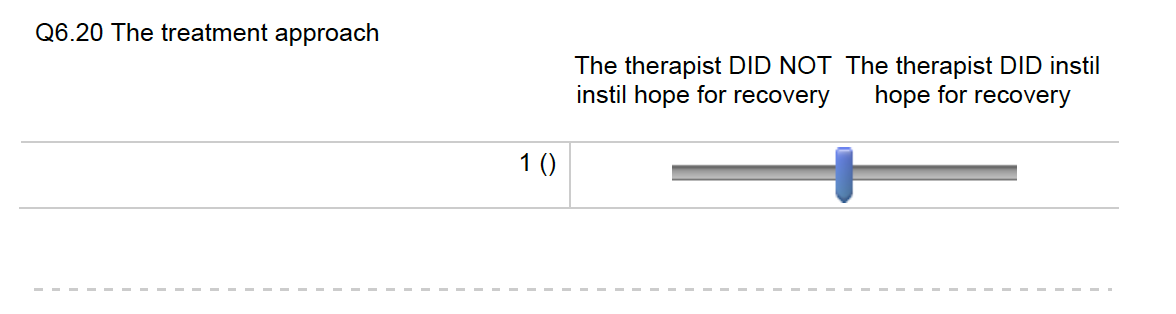


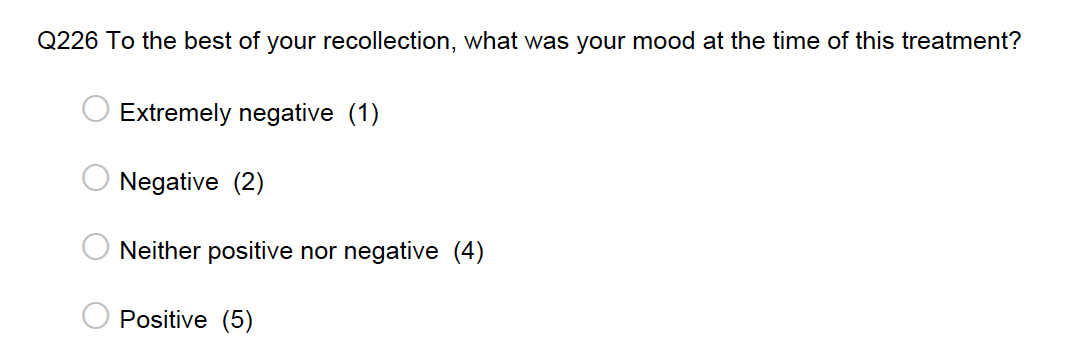

Supplement: Supplementary file 1 — Additional file 1: Appendix. Survey measures. [file 40337_2022_601_MOESM1_ESM.docx]
